# Parents’ Acceptance of Learning about Mindfulness for Managing Pediatric Asthma

**DOI:** 10.3390/children7120262

**Published:** 2020-11-28

**Authors:** Mala Mathur, Karen Pletta, Bradley R. Kerr, Jens Eickhoff, Robin Puett, Megan A. Moreno

**Affiliations:** 1 School of Medicine and Public Health, University of Wisconsin, Madison, WI 53705, USA; karen.pletta@uwmf.wisc.edu (K.P.); bkerr@wisc.edu (B.R.K.); moreno@wisc.edu (M.A.M.); 2 Department of Biostatistics and Medical Informatics, University of Wisconsin, Madison, WI 53726, USA; eickhoff@biostat.wisc.edu; 3 School of Public Health, University of Maryland, College Park, MD 20742, USA; rpuett@umd.edu

**Keywords:** child, asthma, mindfulness, parent

## Abstract

Background: Emerging research suggests mindfulness may reduce stress and asthma symptoms in children, yet there is a gap in understanding parental views towards learning about mindfulness. Objective: This study aimed to compare the perceived acceptance to learn about mindfulness among parents of children with and without asthma, and to understand differences across income levels. Methods: This was a national, cross-sectional, online survey of parents of children 0–18 years. Acceptance was measured with questions on whether parents believe mindfulness could be beneficial while parenting, and if they would be willing to learn about mindfulness. Comparisons of mindfulness acceptance between income level were conducted using chi-square and Fisher’s exact test. Results: Parents of children with asthma were more likely to be interested in learning about mindfulness from their health care provider compared to those without asthma (46% vs. 38%, *p* < 0.0001). At all income levels examined in the study, parents (63–75%) of children with asthma indicated that they agreed or strongly agreed that mindfulness can be beneficial when parenting. Conclusion: Findings suggest an opportunity to incorporate mindfulness teaching into asthma care for pediatric patients of all income levels.

## 1. Introduction

In the U.S., 6 million children have asthma [1] and 49% of children with asthma miss school days due to asthma symptoms [2]. Every year, more than 1 in 6 children with asthma require emergency room visits and more than 1 in 20 children require hospitalization for asthma [1]. It is critical to understand the triggers that exacerbate asthma symptoms to reduce the burden of asthma for families and society.

Several factors can increase the onset and severity of asthma symptoms. First, feeling anxiety and stress can increase a child’s asthma symptoms [3,4,5,6]. Parental stress is also associated with the earlier onset of symptoms of childhood asthma. [7,8]. Increased maternal stress in the postnatal period (first two years of life) has been associated with higher odds of asthma in girls [9]. Paternal post-traumatic stress disorder has been associated with increased asthma symptoms for children at 1 year of age [10]. Economic stressors, such as low income and urban environments, increase childhood asthma symptoms [7,11] and family chaos is associated with higher uncontrolled asthma control for urban minority youth [12]. Lower-income families may already have multiple challenges managing a child with asthma due to socioeconomic factors (income/education), environmental factors (pollutants/allergens), psychosocial stressors (neighborhood violence) and lack of access to medical care and medications [13]. These findings highlight the importance of strategies to reduce anxiety and stress for pediatric asthma patients and their parents especially for lower-income families.

Mindfulness, which can be defined as paying attention on purpose nonjudgmentally [14], and can include such activities as meditation and yoga, may decrease anxiety and stress for children and parents. For children with asthma, yoga interventions may improve lung function testing, decrease asthma symptoms [15,16] and decrease the use of asthma medication [17,18]. Adolescents with higher traits of mindfulness report lower asthma specific stress, better asthma control and increased quality of life [19]. These findings support the utility of mindfulness techniques in reducing stress and helping control pediatric asthma symptoms. In addition to helping children, mindfulness has shown to be associated with lower levels of parenting stress [20]. Parenting stress is associated with poorer psychological adjustment for both caregivers and children with chronic illness [21]. Therefore, efforts to connect pediatric asthma patients and their parents to mindfulness education resources may enhance asthma management and increase family well-being.

In order to provide mindfulness education to families, reduce stress and improve asthma management for children, parents could be key partners. Thus, parent caregiver perceptions of mindfulness are important to know. However, there is a gap in understanding views towards mindfulness and mindfulness education among parents of children with asthma. The purpose of this study was to compare the acceptance of learning about mindfulness between parents of children with and without asthma. Further, given evidence that children from lower-income families may face heightened asthma symptoms, [7,11], a second purpose was to understand the views of parents of children with asthma across income levels.

## 2. Materials and Methods

This national, cross-sectional survey study was part of a larger study focused on parents’ perspectives on pediatric care and took place in October 2018. The University of Wisconsin Education and Social/Behavioral Sciences Institutional Review Board approved this project on 8/16/18 (ID# 2018-1051).

### 2.1. Participants

To recruit parents from throughout the United States, the survey panel platform Qualtrics was used. Previous studies suggest survey panels are an effective approach with broader geographic reach compared to traditional survey approaches [22,23]. In survey panel research studies, prospective participants sign up to be on lists to receive survey invitations. When panelists join Qualtrics, they are asked to complete demographic assessments so that survey invitations can be sent to eligible samples. As an incentive for survey completion, participants earn “Qualtrics Points,” which can be applied to purchases such as gift cards and airline miles.

We requested that Qualtrics recruit a sample with race and ethnicity matching U.S. census data. Since the larger study focused on perspectives of parents with children who are healthy, we also requested a sample including at least 25% of parents with a child with chronic illness. Email invitations were sent to panels of potentially eligible individuals. Prospective participants completed screening questions assessing eligibility for this study: (1) English-speaking, (2) 18 years of age or older and (3) parent of a child under the age of 18.

### 2.2. Measures

Demographic variables included gender, parent age, income, race, ethnicity, education, region of the U.S. and residential community type (i.e., urban, suburban or rural). Participants were asked to indicate whether their child or children have asthma or recurrent wheezing.

The survey additionally assessed three measures of parent views toward mindfulness: (1) whether parents believe mindfulness can be beneficial when parenting, (2) whether parents are interested in learning about how mindfulness can help their child stay healthy, and (3) whether parents would be interested in learning about mindfulness from their healthcare provider. These questions were initially developed by the study team, and then piloted among a group of general pediatricians and parents. Questions were modified based on feedback from pediatricians and parents who completed the pilot. These measures were framed as statements with which participants indicated their agreement on a 5 point Likert scale from “strongly disagree” to “strongly agree”. An option of “don’t know” was also offered.

### 2.3. Analysis

Measures of parent views toward mindfulness were dichotomized as “yes” or “no”. “Agree” or “strongly agree” were coded as a yes while all other responses were coded as no. Analyses excluded for each question, included participants who chose “don’t know”, as the intention was to focus on understanding trends among parents who did and did not show positive views of mindfulness.

The proportions of participants answering “agree” or “strongly agree” to individual items were summarized in frequencies and percentages. Comparisons of the proportions of participants answering “agree” or “strongly agree” to individual items between clinical outcome groups (e.g., asthma vs. no asthma.) were conducted using univariate and multivariate logistic regression analyses. Parent’s age, gender, household income, race, and region of the U.S. were included as covariates in the multivariate analyses. All reported *p*-values are two-sided and *p* < 0.05 was used to define statistical significance. All analyses were conducted using SAS software (SAS Institute Inc., Cary, NC, USA), version 9.4.

## 3. Results

Among the 3000 participants, 87.9% were female, 82.5% were white, 88.7% were non-Hispanic and 69.9% had no college degree. Among all participants, 27% (*n* = 797) had children with asthma. All 50 U.S. states and all 4 regions were represented. See Table 1 for full demographic characteristics of participants.

There was no difference in the belief about mindfulness being beneficial between parents of children with (67.8%, *n* = 754) and without asthma (67.9%, *n* = 2065; *p* = 0.842). Parents of children with asthma (58.3%, *n* = 743) and parents of children without asthma (55.8%, *n* = 2084) did not differ in reporting their interest in learning how mindfulness could keep their children healthy (*p* = 0.311). Parents of children with asthma (48.9%, *n* = 752), however, were more likely than parents of children without asthma (40.6%, *n* = 2083; *p* = 0.0008) to show interest in learning about mindfulness from their health care provider (Table 2).

In all income levels, a sizeable number of parents (63–75%) of children with asthma indicated that they agreed or strongly agreed that mindfulness can be beneficial when parenting. In addition, over half of parents (51–66%) of children with asthma at all income levels showed interest in learning about mindfulness to keep their child healthy, and approximately half (46–55%) indicated interest in learning about this from their health care provider (Table 3).

## 4. Discussion

This cross-sectional study examined beliefs and interest in learning about mindfulness among parents of children with and without asthma. Overall, over two thirds of parents of children with asthma reported that they believed mindfulness could be beneficial for parenting. In general, more parents of children with asthma indicated that they were interested in learning about mindfulness from their health care provider than parents of children without asthma. We also found that among all income levels examined, just over half of parents of children with asthma indicated interest in learning about mindfulness to keep their child healthy.

Over two thirds of parents of children of asthma indicated that mindfulness could be beneficial while parenting. This is important to recognize since caregivers of children who have chronic illness report more stress than caregivers of healthy children [21]. A recent study found that higher parenting stress in parents of children with asthma is associated with more airway inflammation for the child [24], which may in turn affect their child’s asthma symptoms. Thus, teaching mindfulness techniques such as meditation for parents may be a way to reduce stress and improve their child’s asthma control. This study connects what we know about parenting stress for children with asthma and the beliefs that parents have about mindfulness techniques. Understanding this connection paves the way for parents to learn about these techniques as a way to decrease parenting stress, improve their own health and the health of their child.

Parents of children with asthma in this study were more likely to report that they were interested in learning about mindfulness and learning about it from their health care provider compared with parents of children without asthma. Since children with asthma may have frequent visits with multiple health care providers, it is possible their parents are more receptive to learning about mindfulness as they may be accustomed to receiving information from health care providers on how to manage their child’s condition. This study highlights that parents of children with asthma may be particularly receptive to learning about mindfulness, which may allow for referral of patients to mindfulness resources through the health care system and within the community. These resources may represent a low-cost way to improve pediatric asthma care that can be directed at children/adolescents or parents. Further studies are needed to explore how parents would like to learn about mindfulness skills such as meditation and yoga, and if learning about mindfulness practices results in actual implementation of these practices.

Physicians may expect that lower income families could be less receptive to education about mindfulness due to a lack of resources. However, our study suggests that many parents may be willing to learn mindfulness techniques, regardless of income. This opens up a promising opportunity to help families who may particularly benefit from addressing stress that can trigger asthma symptoms. This may be especially helpful for lower income families where there may be increased stress and the burden of asthma is higher [13]. It may be helpful for providers to know about and provide free or low cost resources such as videos, podcasts or community programs to support these families’ interests.

Limitations of this study include participants who were predominantly white, female and without a college degree. In addition, the survey participants were all English speaking and had access to the internet to complete this survey. Future studies should examine the perspectives of families from minority and non-English speaking families. In addition, future studies could look at the baseline level of engagement in mindfulness practices to better understand parents’ perspectives on learning more about mindfulness practices. In our study, participants with children with asthma included children with asthma or recurrent wheezing, and findings may not be specific to families with asthma alone. However, it may be that these patients have similar symptoms and similar healthcare experiences.

## 5. Conclusions

In this study, parents of children with asthma in all income levels indicated that mindfulness could be beneficial for parenting, and just over half showed interest in learning about mindfulness from their health care provider. With the emerging research showing benefits of mindfulness on stress and asthma, coupled with the interest of parents in learning about mindfulness, health care providers may have an opportunity to address stress for both parents and children/adolescents. Mindful interventions may be specifically targeted to families of children with asthma since promoting mindfulness techniques may reduce asthma symptoms and improve asthma control.

## Figures and Tables

**Table 1:** Demographic Characteristics of Parent Participants (*n* = 3000).

Gender	
Female	2621 (87.9%)
Male	360 (12.1%)
Parent Age	
<20 years	14 (0.6%)
20–30 years	550 (21.7%)
30–40 years	1059 (41.7%)
>40 years	915 (36.1%)
Income	
<$50,000	1410 (47.2%)
$50,000–100,000	856 (28.6%)
>$100,000	724 (24.2%)
Race	
White	2466 (82.5%)
Black	266 (8.9%)
Other	167 (5.6%)
Asian	90 (3.0%)
Ethnicity	
Non-Hispanic	2645 (88.7%)
Hispanic	338 (11.3%)
Education	
No college degree	2093 (69.9%)
College degree	900 (30.0%)
Region	
Midwest	756 (25.6%)
Northeast	518 (17.6%)
South	1199 (40.6%)
West	477 (16.2%)
Location	
Rural	1050 (35.3%)
Suburban	1289 (43.3%)
Urban	634 (21.3%)

**Table 2:** Parents of children with and without asthma who indicated (agree or strongly agree) acceptance of learning about mindfulness.

Parents of Children	Belief That Mindfulness Can Be Beneficial While Parenting	Adjusted Comparison *p* Value *	Interest in Learning about Mindfulness to Keep Their Child Healthy	Adjusted Comparison *p* Value *	Interest in Learning about Mindfulness from Their Health Care Provider	Adjusted Comparison *p* Value *
With Asthma 797 (27%)	754 (67.8%)	0.842	743 (58.3%)	0.311	752 (48.9%)	0.0008
Without Asthma 2203 (73%)	2065 (67.9%)		2084 (55.8%)		2083 (40.6%)	

* Adjusted by parent age, gender, race, region and household income.

**Table 3:** Endorsement of mindfulness (agree/strongly agree) by parents of children with asthma by income level.

Parent Income Level	Belief That Mindfulness Can Be Beneficial While Parenting	Interest in Learning to Keep Child Healthy	Interest in Learning from Their Health Care Provider
<$50,000	345 (63.2%)	340 (51.2%)	344 (44.5%)
$50,000–$100,000	197 (68.5%)	193 (62.7%)	193 (50.3%)
>$100,000	211 (74.9%)	209 (66.0%)	214 (55.1%)
